# A 5-year comprehensive evaluation of maxillofacial injuries in polytrauma patients at a tertiary hospital – an epidemiological study

**DOI:** 10.2340/aos.v83.40250

**Published:** 2024-04-10

**Authors:** Pradeep Muralidhar, Vinod Bandela, Abdul Ahad Ghaffar Khan, Shahi Jahan Shah, Abosofyan Salih Atta Elfadeel, Ram B Basany, Devi Singh Amgoth, Shilpa Param

**Affiliations:** a Department of Dentistry & Maxillofacial Surgery, Apollo Institute of Medical Sciences and Research, Hyderabad, Telangana, India; b PhD Scholar, Saveetha University, Chennai, India; c Department of Prosthetic Dental Sciences, College of Dentistry, Jouf University, Sakaka, Saudi Arabia; d Department of Oral & Maxillofacial Surgery, College of Dentistry, King Khalid University, Abha, Saudi Arabia; e Department of Prosthodontics, SVS Institute of Dental Sciences, Mahabubnagar, Telangana, India; f Maxillofacial Surgeon, Government General Hospital, Nizamabad, Telangana, India; g Dental Surgeon, Apollo Hospitals, Hyderabad, Telangana, India

**Keywords:** Polytrauma, maxillofacial injuries, facial fractures, accidents, maxillofacial surgeon

## Abstract

**Objectives:**

To report and analyze the pattern of maxillofacial injuries in trauma victims and to define the role of a maxillofacial surgeon in an emergency trauma care team.

**Materials and methods:**

Trauma patients reported and reporting to the casualty of a tertiaryhospital with facial injuries and other suspected concomitant injuries in the body were included in this study. The complete medical and radiographic records of each patient were reviewed and data was collected in a standard proforma in this 5-year clinical study (3 year of retrospective and 2 year of prospective study). The complete data related to the facial injuries and associated systemic trauma was recorded and statistical analysis conducted.

**Results:**

A total of 18,369 patients with trauma were admitted to the hospital from May 2018 to April 2023. Out of these, 11,277 (61.4%) were males and 7,092 (38.6%) were females. Seventy percent of the reported cases with trauma were in the age group of 14–40 years. The incidence of trauma during the monsoon season was highest (*n* = 7,927, 43%). The commonest etiological factor leading to trauma was road traffic accident (*n* = 4,510, 40%). Among facial injuries, the mandibular fractures (*n* = 1,821, 41%) were predominant.

**Conclusions:**

The management of polytrauma patients should be undertaken by a team of specialists which should include a maxillofacial surgeon as facial injuries were common. This data is essential in developing and assessing the preventative strategies aimed at decreasing the frequency of facial and other injuries.

## Introduction

Clinical studies and reviews are valuable scientific tools as these significantly contribute to the scientific literature in understanding the disease frequency and patterns. Comparison of such data with regional variations will help in proposing custom-made treatment strategies, which might suit the given population. These studies form a cornerstone and pave a path for initiating any advanced research programs in understanding various diseases. Many such studies about the pattern of maxillofacial injuries have been reported and published from different countries, but the demographic data is difficult to estimate as there are many variables [1–7].

Facial fractures vary according to the type, cause of injury, severity, and based on the population on which the study is undertaken. Because of the prominent and exposed position, a considerable percentage of trauma victims experience maxillofacial fractures that have been on the rise in recent decades. The most common facial injuries are mandibular fractures that account for the majority of traumas and rank as the tenth most frequent bone-injury in the body and the second most frequent facial bone injury that requires treatment by a maxillofacial surgeon. Nasal bones are more often affected as a result of facial trauma [8]. Cranio-maxillofacial injuries affect a significant proportion of trauma patients. They can occur in isolation, or in combination with other injuries including cranial, spinal, upper, and lower body [9–12].

The purpose of this study was to analyze the type of maxillofacial injuries and the factors influencing their distribution in and around Mahabubnagar province of Telangana state. Our goal was to analyze facial trauma by evaluating the patients’ data regarding dento-alveolar and facial bone fractures, soft tissue injuries and to investigate the five main causes of facial injuries. Furthermore, this study assessed the statistical tendencies of cranio-maxillofacial trauma concomitant with other injuries in the body and analyzes such patterns, their treatment priorities, and to define the role of maxillofacial surgeon in polytrauma patients.

## Materials and methods

This study was conducted at the Department of Oral & Maxillofacial surgery in a tertiary hospital of Mahabubnagar district of Telangana. A convenient sample was chosen from the available data. All trauma patients with facial injuries and other suspected concomitant injuries in the body and patients with facial injuries reporting to the tertiary hospital were included in the study. The complete medical and radiographic records of each patient were reviewed and data was recorded in a standard case sheet proforma. This is a unique 5-year epidemiological study, analyzing the data from May 2018 to April 2021 retrospectively (3 years) and prospectively from May 2021 to April 2023 (2 years).

The facial injuries were identified according to their anatomical location, age, sex of the patient, cause of injury, additional fractures, and associated systemic injuries. Anatomical classification of the mandibular fractures was adopted from the system described by Ivy RH & Curtis L, midface fractures from the system introduced by Le Fort, and zygomatic complex fractures as proposed by Rowe and Killey. Parameters used for statistical analysis in the study are gender of the patient, age groups, seasonal distribution of trauma, place of occurrence of trauma, history of alcohol intake, etiology, facial injuries, and other concomitant injury.

### Statistical analysis

The data was entered in MS Excel (2016 version), checked, and verified for errors. Statistical analysis was conducted using the Mann–Whitney U test, Kruskal–Wallis test, and Chi-square test. Probability value of 0.05 or less was considered to be statistically significant.

## Results

### Demographic details

A total of 18,369 patients reported with trauma from May 2018 to April 2023, out of which 11,277 (61.4%) were males and 7,092 (38.6%) were females presenting a ratio of 1.5:1 (Table 1).

**Table 1:** Demographic data.

Gender	*n*	%
Male	11,277	61.4
Female	7,092	38.6
Total	18,369	100.0

The total sample size was divided according to the age of incidence of cranio-facial injuries. The highest incidence of trauma (*n* = 12,891, 70%) was found in the age group of 14–40 years and least was found (*n* = 106, 0.6%) in the age group of 0–6 years. There was no statistically significant difference (*p* = 0.686) between male and female samples for different age groups (Table 2).

**Table 2:** Age-wise incidence of cranio-facial injuries.

Age	Male		Female		Total	
	*n*	%	*n*	%	*n*	%
Infancy (birth – 6 years)	71	0.4	35	0.2	106	0.6
Young (6 years–14 years)	912	5	567	3.1	1,479	8.1
Adult (14 years–40 years)	7,927	43	4,964	27	12,891	70
Geriatric (>40 years)	2,367	13	1,526	8.3	3,893	21.3
Total	11,277	61.4	7,092	38.6	18,369	100

The most common etiological factor behind trauma was found to be Road Traffic Accidents (RTA) (*n* = 6,711, 37%) with predominance among males (*n* = 4,510, 40%). It was observed that assaults were more commonly seen in the female population (*n* = 2,127, 30%), whereas males dominated in sports and occupation related injuries (12% and 6%) (Table 3).

**Table 3:** Etiology of cranio-facial injuries among males and females.

Etiology	Male		Female		Total	
	*n*	%	*n*	%	*n*	%
Motor vehicle/cycle/three wheeler	4,510	40	2,201	31	6,711	37
Assault	2,822	25	2,127	30	4,949	27
Sports	1,353	12	638	9	1991	11
Occupational	676	6	283	4	959	5
Falls	1,691	15	1,418	20	3,109	17
Others	225	2	425	6	650	4
Total	11,277	100	7,092	100	18,369	100.0

The main cause of facial injuries was RTA (*n* = 1,134, 42%), where the mandible is the most commonly fractured bone (45%) out of all the causes of injuries. Zygoma fractures were commonly seen with falls (39.9%). Mandible fractures predominated in occupational and sports related injuries (Table 4).

**Table 4:** Etiology of cranio-facial injuries and their anatomical distribution.

Facial fracture	Maxilla		Mandible		Zygoma		Total	
	*n*	%	*n*	%	*n*	%	*n*	%
Motor vehicle/cycle	86	51	841	45	207	29.9	1,134	42
Assault	36	22	280	15	108	15.6	424	16
Sports	4	2	93	5	1	0.4	98	4
Occupational	5	3	56	3	3	0.4	64	2
Falls	28	17	373	20	276	39.9	677	25
Others	8	5	224	12	97	14	329	12
Total	167	100	1,867	100	692	100	2,726	100

A higher incidence of trauma was observed during the monsoon (July–October) when compared to summer (March–June) and winter (November–February) for the entire study period which was statistically significant (*p* = 0.016) (Table 5).

**Table 5:** Seasonal distribution of cranio-facial injuries over the study period.

Seasonal distribution	2018		2019		2020		2021		2022		2023	
	*n*	%	*n*	%	*n*	%	*n*	%	*n*	%	*n*	%
Summer (March–June)	265	15	558	20	667	25	844	20	1,028	20	1,184	64
Monsoon (July–October)	1,006	56	1,371	50	1,154	44	1,902	45	2,573	50	0	0
Winter (November–February)	514	29	826	30	805	31	1,478	35	1,542	30	652	36
Total	1,785	100	2,755	100	2,626	100	4,224	100	5,143	100	1,836	100

As the hospital is very close to the national highway, results revealed that the number of accidents in the case of male patients doubled (66%) as compared to females (30%) because males suffered injuries under the influence of alcohol (97.9%) and in the case of females this value was only 2.1%. This shows that there was significant difference between the location of the accident in the highway and city/village/towns and the influence of alcohol played a significant role in males (Table 6).

**TAble 6:** Location of cranio-facial injuries and History of alcohol intake.

Location of the accidents	Male		Female		Total	
	*n*	%	*n*	%	*n*	%
City, town, village	1,534	34	1,985	70	3,519	48
Highway	2,976	66	851	30	3,827	52
Total	4,510	100	2,836	100	7,346	100
History of alcohol intake	3,847	97.9	81	2.1	3,928	100

### Anatomic distribution of the fractures

The significant finding in this study was that the facial fractures (*n* = 4,446, 28%) took second place after orthopedic injuries (*n* = 6,116, 39%) with a high proportion of male patients and least cases were reported related to pulmonary injuries (2%) (Table 7).

**Table 7:** Concomitant injuries among males and females.

Concomitant injury	Male		Female		Total	
	*n*	%	*n*	%	*n*	%
Neurological	1,127	11.8	542	9	1,669	11
Abdominal	905	9.4	422	7	1,327	8
Pulmonary	225	2.3	63	1	288	2
Opthalmological	563	5.9	1,205	20	1,768	11
Orthopedic	3,946	41.2	2,170	36	6,116	39
Facial	2,819	29.4	1,627	27	4,446	28
Others	1,692	-	1,063	-	2,755	-
Total	11,277	100	7,092	100	18,369	100

Among facial injuries (*n* = 4,442), mandibular fractures accounted for the maximum frequency (*n* = 1,821, 41%) followed by dento-alveolar fractures (*n* = 965, 22%). Soft tissue injuries like abrasions and lacerations constituted 19% (*n* = 855) of the total sample size followed by zygomatic and naso-orbito-ethmoid (NOE) fractures (*n* = 592, 13%) and midface fractures involving occlusion (*n* = 167, 4%) (Table 8).

**Table 8:** Occurrence of facial injuries among males and females.

Facial injury	Male		Female		Total	
	*n*	%	*n*	%	*n*	%
Abrasions and laceration	594	21.1	261	16.1	855	19
Dental trauma/dento-alveolar fracture	657	23.3	308	19	965	22
Maxillary	126	4.5	41	2.5	167	4
Mandibular	1,070	38	751	46.3	1,821	41
Zygomatic complex & NOE	338	12	254	15.7	592	13
Frontal bone	34	1.2	8	0.5	42	1
Total	2,819	100	1,623	100	4,442	100

Among mandibular fractures, the highest incidence was found to be in the parasymphysis (*n* = 614, 33.7%) followed by angle of the mandible (*n* = 386, 21.2%) and the least cases suffered coronoid fractures (*n* = 5, 0.3%). A total of 50% (*n* = 83) of midface fractures involving occlusion were of Le Fort type I, 29% (*n* = 49) were of Le Fort type II, and 21% (*n* = 35) were of Le Fort type III. (Tables 9 and 10)

**Table 9:** Anatomical occurrence of mandibular fractures among males and females.

Mandibular fracture	Male		Female		Total	
	*n*	%	*n*	%	*n*	%
Symphysis	208	19.4	139	18.5	347	19.1
Parasymphysis	292	27.3	322	42.9	614	33.7
Body	162	15.1	39	5.2	201	11.0
Angle	214	20	172	22.9	386	21.2
Condyle	189	17.7	79	10.5	268	14.7
Coronoid	5	0.5	0	0	5	0.3
Total	1,070	100	751	100	1,821	100

**Table 10:** Incidence of maxillary fractures among males and females.

Maxillary fracture	Male		Female		Total	
	*n*	%	*n*	%	*n*	%
Le Fort type I	67	53	16	39	83	50
Le Fort type II	38	30	11	27	49	29
Le Fort type III	21	17	14	34	35	21
Total	126	100	41	100	167	100

Of the 587 zygomatic complex and NOE fractures, type II fractures were the most common (*n* = 155, 26%) and type III fractures were least common (*n* = 13, 2%). Male predominance was seen in the above-mentioned category of fractures (Table 11).

**Table 11:** Occurrence and type of zygomatic complex and NOE fractures.

Zygomatic complex & NOE	Male		Female		Total	
	*n*	%	*n*	%	*n*	%
Type I	67	20.1	54	21.3	121	21
Type II	84	25.2	71	28	155	26
Type III	6	1.8	7	2.8	13	2
Type IV	10	3	14	5.5	24	4
Type V	62	18.6	23	9.1	85	14
Type VI	48	14.4	43	16.9	91	16
Type VII	40	12	32	12.6	72	12
Type VIII	16	4.8	10	3.9	26	4
Total	333	100	254	100	587	100

### Frequency of associated injuries

The incidence of facial fractures in polytrauma patients after analysis revealed that facial fractures are more frequently seen along with head injury (45%) as compared to orthopedic, abdominal, pulmonary, and ophthalmological injuries. Fifty-seven per cent of intracranial bleeds were associated with facial bone fractures of which 85% were zygomatic complex fractures. Twenty-five per cent of ophthalmological injuries were associated with facial bone fractures of which 44.3% were zygomatic complex fractures. Seventy-one per cent of these patients presented with clinical signs of sub-conjunctival hemorrhage. Another significant finding in the outcome was the loss of vision seen in four individuals with zygoma fractures out of which three patients had retro bulbar hemorrhage at the time of initial presentation (Table 12).

**Table 12:** Occurrence of zygomatic complex and NOE fractures among males and females.

Injuries	Maxilla		Mandible		Zygoma		Total	
	*n*	%	*n*	%	*n*	%	*n*	%
Neurological injures	107	54.31	32	26	73	49.0	212	45
Abdominal injury	0	0.00	4	3	0	0.0	4	1
Pulmonary injury	2	1.02	5	4	1	0.7	8	2
Opthalmological injuries	44	22.34	8	7	66	44.3	118	25
Orthopedic injuries	44	22.34	73	60	9	6.0	126	27
Total	197	100	122	100	149	100	468	100

### Outcome of the injuries

Of all 18,369 patients there were 3,893 patients who presented with various kinds of morbidity (21.1%) and the mortality rate of 0.5% (*n* = 102) patients was mostly associated with severe head and abdominal injuries (Table 13).

**Table 13:** Outcome of injuries.

Outcome of injuries	*n*	%
Morbidity	3,893	97.4
Mortality	102	2.6
Total	3,995	100

## Discussion

Understanding the patterns, etiology, and consequences of trauma is indispensable in estimating the frequency of a disease and finding its association suggests a potential cause of a disease. By doing so, it is possible to recommend a strategy for injury prevention and effective use of medical resources. The first step for better understanding of the health issues in a particular demographic is to conduct descriptive research. This study is unique in the fact that it is the first of its kind originating from this geographic area.

One or more of the bones, including the maxillae, zygomatic, nasal, frontal, and ethmoidal bones, as well as the bony components of the orbits and anterior fossa of the skull, may be involved in middle-third facial fractures. Damage to these bones in high-speed impacts may result in over 50 fragments; it becomes impossible to consider reducing them completely and restoring all the minute details of the contour [13].

Mandibular fractures are the commonest facial injuries constituting the bulk of the trauma and can cause various impairments. This includes temporomandibular joint pain, disturbed occlusion, poor masticatory efficiency, disorders of salivary origin, obstructive sleep apnoea, and chronic pain. They can alter facial development resulting in disfiguring debility among children [3].

A total of 18,369 patients reported to the hospital with trauma from May 2018 to April 2023 over a period of 5 years. Out of these, 11,277 (61.4%) were males and 7,092 (38.6%) were females presenting a ratio of 1.5:1. The findings of male predominance were similar to the results of other studies of facial bone fractures. The majority of fractures were found among males (almost 85%), probably due to their higher involvement in physical activity which could result in more RTA or altercations [8, 9, 14]. Boffano et al. in their systematic studies found that men outnumbered women with the ratio being more than 2:1 for maxillofacial trauma [15].

It was observed during the study period of 5-years that the annual incidence of trauma has increased by an average of 6.99%. The frequency of incidence of trauma appears to be highest during the months of monsoon compared to the other two seasons. These findings are similar to that of research conducted by the crime department of the District of Columbia (uniform crime reports), 1995–2000. In a study, it was reported that maxillofacial injuries occurred mostly during weekends and maximum number of cases occurred in the months of August and September [16]. These results substantiate the fact that the rainy and holiday seasons are associated with a higher risk of facial bone fractures. Similar findings were also reported in other studies [1, 11, 17].

The etiology of face injuries found in this study is consistent with findings from other Middle East countries. [2, 18–20]. Our findings can also be compared to the research conducted in other regions of the world. Assault-related maxillofacial injuries were reported to be more common in developed countries. In our study, assault-related injuries accounted for 25% of the total cases.

In our study, influence of alcohol contributed not only to the increase in violence, but was also an important factor contributing to facial and other concomitant fractures that occurred in motor vehicle accidents which are in accordance with other studies. In spite of enforcement of strict rules against drunken driving, our study reported 40 and 2.1% incidence of facial trauma among males and females under the influence of alcohol [9, 21, 22].

This data indicates that the facial bones have very low tolerance to impact forces, the nasal bones being the least resistant, followed by the zygomatic arch. The mandibular body and ramus are more sensitive to lateral impacts than the symphysis, and the maxilla is extremely sensitive to horizontal impacts [13].

Out of 4,446 facial injuries, the most common were mandibular fractures followed by dental trauma/dento-alveolar fracture. This is in accordance with other studies published in the literature where it was stated that the mandible is the most commonly fractured bone in the face followed by maxilla and zygoma [22]. However, some authors state that the nasal bone is the most commonly affected [8]. In another study, the most frequently observed fracture involved the mandible followed by orbital-zygomatic-maxillary fractures [23].

Among mandibular fractures, the parasymphysis was the most common site to be fractured followed by angle, symphysis, condyle, body, and coronoid. Ellis et al., in their retrospective study on 4,711 trauma patients, found a 45.4% incidence of mandibular fractures, which was more than any other facial bone fractures [24]. In a study conducted by Fasola et al. It was shown that mandibular fractures made up nearly three quarters of the total number of fractures [25].

Among the midface fractures involving dental occlusion, Le Fort I fractures were found to be the most frequent and among those not involving dental occlusion displaced isolated zygomatic arch fractures were most frequent. Though Le Fort I fractures are uncommon among facial fractures, these are more common in the midface region in this study. Brucoli et al. found that the most frequent cause of zygomaticomaxillary complex fractures was assault followed by falls [26].

The commonly associated injuries with facial trauma noted in our study were neurological injuries, followed by orthopedic, ophthalmological, pulmonary, and abdominal injuries. Of all the concomitant injuries with facial injuries among polytrauma victims, neurological injuries were followed by soft tissue injuries.

In this combined retrospective and prospective study of 18,369 patients, it was evident that there was high prevalence of maxillofacial injuries among young adults and increased prevalence of RTA during monsoon and vacation seasons. Assaults in females were significant.

While the role of maxillofacial surgeons in trauma care is widely recognized, our study focused on the unique characteristics of maxillofacial injuries in our specific region. The regional variability in etiology and patterns of trauma highlights the necessity of understanding local epidemiology for effective patient care. Despite the general awareness of the maxillofacial surgeon’s role, the specific challenges and opportunities presented in our region warrant a tailored approach to patient management. This study, therefore, contributes not only to the academic literature but also provides actionable insights for healthcare practitioners and policymakers involved in regional trauma care.

## Clinical implications

The findings of this study have direct implications for clinical practice in our region. By recognizing the predominant causes and patterns of maxillofacial injuries, healthcare professionals, especially maxillofacial surgeons, can refine their approaches to patient care. The data presented here serve as a practical guide for developing targeted intervention strategies, improving emergency trauma care protocols, and enhancing the overall efficiency of the healthcare system in addressing maxillofacial trauma in our community.

## Conclusion

In conclusion, our 5-year epidemiological study highlights the pivotal role of maxillofacial surgeons in polytrauma management, particularly concerning facial injuries prevalent in RTAs. The study underscores the need for a collaborative approach in trauma care teams, emphasizing timely intervention to prevent facial deformities and associated functional issues. The key takeaways include advocating for multidisciplinary teams, implementing targeted preventive strategies focused on road safety, intensifying educational campaigns against alcohol-related incidents, and recognizing the significance of public awareness, especially among the younger population. While reaffirming the common association of facial trauma with RTAs, the study serves as a foundation for informed decision-making in healthcare policies and trauma care protocols. Further research is encouraged to explore nuanced aspects of injury prevention and treatment modalities.
